# The Causes and Treatment of Goitre

**Published:** 1882-01

**Authors:** 


### THE CAUSES AND TREATMENT OF GOITRE.

Mr. G. A. Lebour, M. A., Professor of Geology in the University of Durham College of Physical Science, Newcastle-on Tyne, has published a brief paper on the geological distribution of endemic goitre in England. He first examines the data obtained on the subject from carefully conducted studies in France, and next considers the distribution of the disease in England.

"In England, as in France, one point," he says, "and one alone, seems to be established as being common to those rocks on which goitre is found not to occur; the absence of limestone, together with that of metallic impurities, and especially sulphide of iron. In both countries the rocks which support most goitre are such as are both calcareous and metalliferous. But there are plenty of facts to show that metalliferous impurities alone cannot be credited with the origin of the disease, else the Devonian and the granite would not be free from it. Neither will the absence of limestone alone be sufficient to check the growth of bronchocele, else the lignitiferous beds of France and the ferruginous sands of the weald would not support it. On the whole, there is a striking sameness in the geological distribution of the disease in the two countries."

Dr. Edward Woakes, in the *Lancet*, March, 1881, gives the results of his experience of *fluoric acid* in the treatment of goitre. Of twenty cases, seventeen were cured. The treatment extended over a lengthened period. Fluoric acid is not to be regarded as a specific, but rather as a basis of constitutional treatment, with which iron, in anæmic subjects, may be advantageously combined, while, coincidentally with its internal administration, recourse may be had to such local procedures as experience has shown to favor dispersion of goitre. In many cases, the effect of the fluoric acid was immediate. In one case, a large fibrous goitre that had existed for two years was markedly diminished in a week, by the use of half drachm doses of the acid, three times a day. In other cases, improvement goes on up to a certain point, and then the tumor remains stationary. Then an injection of iodine into the substance of the tumor causes absorption to recommence, and often it is only necessary to continue with the acid, or perhaps to employ a second injection. The rationale of the *modus operandi* of fluoric acid, is that it restores the lost contractile function of the impoverished ganglia that supply the *nervi vasorum*, either by acting as a physiological spur to their function, or by furnishing a deficient element in their composition, or without which the nerve currents generated in them are reduced to *nil.*

A case of extirpation of a colloid goitre is reported by Dr. Richelat in the *Annales des Mal. du Larynx*, Dec. 1880. The gland was removed through a curved incision with its convexity downwards, extending to the common carotid on each side, and passing immediately above the sternal notch in the median line. The trachea was found strongly curved to the right, and slightly flattened from before backward, but not softened or degenerated. It was intimately adherent to the gland, from which it was removed from below upwards. There was very little loss of blood during the operation. Silk ligatures, which were first employed, were mostly replaced by catgut ones, and the wound was united by wire sutures. There was severe dysphagia for several days, followed at the end of a week by an attack of bronchitis and tracheitis. The result was complete cicatrization of the wound in the fifth week, accompanied by complete and persistent aphonia.

In Geneva, where goitre is very common, its surgical extirpation is comparatively frequent. It is stated in a report of the Cantonal Hospital, in the *Lancet*, June 11th, 1881, that Professor Reverdin has operated thirteen times for the extirpation of goitre, and Dr. Auguste Reverdin, four times. Out of these seventeen cases three have died—viz., one case of calcified, suffocating goitre; one case of ordinary suffocating goitre; and one case of cancer of thyroid, with thrombosis of jugular vein. Out of the remaining fourteen operations, twelve have healed by first intention, sometimes with little fistulæ lasting for a time, and two have healed by second intention.—*Med. and Surg. Reporter.*

